# L-type calcium channel blockers and substance P induce angiogenesis of cortical vessels associated with beta-amyloid plaques in an Alzheimer mouse model

**DOI:** 10.1016/j.neurobiolaging.2014.12.027

**Published:** 2015-03

**Authors:** Nina Daschil, Kathrin M. Kniewallner, Gerald J. Obermair, Birgit Hutter-Paier, Manfred Windisch, Josef Marksteiner, Christian Humpel

**Affiliations:** a Department of Psychiatry and Psychotherapy, University Clinic of General and Social Psychiatry, Medical University of Innsbruck, Innsbruck, Austria; b Department of Physiology and Medical Physics, Medical University of Innsbruck, Innsbruck, Austria; c QPS Austria GmbH, Grambach, Austria; d NeuroScios GmbH, Graz, Austria; e Department of Psychiatry and Psychotherapy A, General Hospital LKH Hall, Hall, Austria

**Keywords:** Alzheimer, Beta-amyloid plaque, Reactive astrocytes, Vessels, L-type calcium channel, Substance P, Angiogenesis

## Abstract

It is well established that L-type calcium channels (LTCCs) are expressed in astroglia. However, their functional role is still speculative, especially under pathologic conditions. We recently showed that the α_1_ subunit-like immunoreactivity of the Ca_V_1.2 channel is strongly expressed in reactive astrocytes around beta-amyloid plaques in 11-month-old Alzheimer transgenic (tg) mice with the amyloid precursor protein London and Swedish mutations. The aim of the present study was to examine the cellular expression of all LTCC subunits around beta-amyloid plaques by in situ hybridization using ^35^S-labeled oligonucleotides. Our data show that messenger RNAs (mRNAs) of the LTCC Ca_V_1.2 α_1_ subunit as well as all auxiliary β and α_2_δ subunits, except α_2_δ-4, were expressed in the hippocampus of age-matched wild-type mice. It was unexpected to see, that cells directly located in the plaque core in the cortex expressed mRNAs for Ca_V_1.2 α_1_, β_2_, β_4_, and α_2_δ-1, whereas no expression was detected in the halo. Furthermore, cells in the plaque core also expressed preprotachykinin-A mRNA, the precursor for substance P. By means of confocal microscopy, we demonstrated that collagen-IV-stained brain vessels in the cortex were associated with the plaque core and were immunoreactive for substance P. In cortical organotypic brain slices of adult Alzheimer mice, we could demonstrate that LTCC blockers increased angiogenesis, which was further potentiated by substance P. In conclusion, our data show that brain vessels associated with beta-amyloid plaques express substance P and an LTCC and may play a role in angiogenesis.

## 1 Introduction

Progressive impairment in memory and cognition is a key clinical feature of Alzheimer's disease (AD). The disorder is morphologically characterized by the extracellular deposition of β-amyloid (Aβ), intraneuronal tau pathology, synaptic loss, neuronal cell death, Aβ angiopathy, and inflammatory processes. Besides these hallmarks of AD, altered Ca^2+^ regulation is likely to play an important role in AD pathology (Thibault et al., 2007). It has been suggested that increased activity of L-type calcium channels (LTCCs) drives many of the markers of pathology in aging and AD brains. LTCCs consist of a pore-forming α_1_ subunit (Ca_V_1.1, Ca_V_1.2, Ca_V_1.3, or Ca_V_1.4) and accessory α_2_δ and β subunits (Arikkath and Campbell, 2003, Catterall, 2000, Striessnig et al., 2006). Although α_1_ subunits form the proper channel pore and contain the voltage sensor, they also exhibit pharmacologic properties. On the other hand, the accessory subunits regulate membrane expression and gating properties of the Ca^2+^ channels (Obermair and Flucher, 2013). These voltage-gated Ca^2+^ channels provide a major mechanism for activity-induced Ca^2+^ entry into neurons and regulate multiple neuronal cell functions, including excitability, transcriptional regulation, and synaptic plasticity. Accordingly, aberrant activity of LTCCs and dysregulation of Ca^2+^ homeostasis might be involved in AD pathology (Green et al., 2007, Santos et al., 2010).

Transgenic mice overexpressing human amyloid precursor protein (APP) with mutations that predispose for familial AD, provide potent models for testing specific alterations associated with the upregulation of Aβ (Crews et al., 2010, Rockenstein et al., 2007). In recent studies, we demonstrated that intense Ca_V_1.2 α_1_-subunit-like immunoreactivity (-LI) is found in reactive astrocytes of 11-month-old transgenic (tg) mice overexpressing human APP751 with the London (V717I) and Swedish (K670M/N671L) mutations (Daschil et al., 2013, Willis et al., 2010). However, at present it is unclear whether the intense staining of Ca_V_1.2 α_1_-subunit-LI correlates with subunit messenger RNA (mRNA) expression in the same cells. Up to now, it is unknown how the increased availability of astroglial Ca_V_1.2 α_1_-subunits is related to AD pathogenesis. Furthermore, it is unclear whether this astroglial calcium channel indeed represents a functional channel. We recently showed the absence of the β_4_ subunit in these reactive astrocytes around plaques (Daschil et al., 2013). Complete immunocytochemical localization of LTCC subunits is not possible due to the lack of immunohistochemistry-specific antibodies.

This study focused on the localization and expression of of β and α_2_δ subunits using in situ hybridization. For that purpose 11-month-old wt and tg mice overexpressing human APP with the London and Swedish mutations (APP_SL_) were used. Our analysis focused on the localization of mRNAs in association with Aβ plaques. We compared the expression pattern with that of preprotachykinin-A (PPT-A) mRNA (the precursor for substance P, SP), because this peptide has been shown to be localized in reactive astrocytes around plaques. We show that although Ca_V_1.2 α_1_-LI is strongly expressed in reactive astrocytes around plaques, its mRNA is not detectable. It was unexpected to find cells expressing the LTCC subunit and PPT-A mRNAs directly above the plaque core in the cortex. Using confocal microscopy, we demonstrate that brain vessels are associated with Aβ plaques and express substance P and an LTCC. We could demonstrate that LTCC blockers induced angiogenesis of vessels in close proximity to Aβ plaques in cortical organotypic brain slices. This effect was potentiated by the addition of substance P.

## 2 Methods

### 2.1 Animals

Transgenic male animals overexpressing human APP751 with the London (V717I) and Swedish (K670M/N671L) mutations under the regulatory control of the neuron-specific murine Thy-1 promoter (mThy-1-hAPP751), heterozygous with respect to the transgene, on a C57BL/6 background were used (Havas et al., 2011, Hutter-Paier et al., 2004). The colony was sustained by crossing APP_SL_ mice and C57BL/6 mice (Harlan Winkelman, Germany). Age-matched littermates (wt) were used as controls. All mice were housed according to standard animal care protocols, fed regular animal diet ad libitum, and maintained in a pathogen-free environment in individual ventilated cages at QPS Austria (Grambach, Austria). The transgenic status of each animal was confirmed by polymerase chain reaction (PCR) of tail snips using specific primers and the appropriate hybridization probe. The age of the animals at sacrifice was 11 months. All experiments were conducted in accordance with the relevant Austrian Animal Protection and Welfare Act.

For organotypic brain slices wt (C57BL/6N) and tg APP_SDI (expressing APP and harboring the Swedish K670N/M671L, Dutch E693Q, and Iowa D694N mutations, C57BL/6-Tg [Thy1-APPSwDutIowa] BWevn/Mmjax) mice were purchased from The Jackson Laboratory and housed at the Medical University of Innsbruck animal facility, providing open access to food and water under 12 hour-light and 12-hour-dark cycles. The mice have been generated and extensively characterized by Davis et al. (2004).

### 2.2 In situ hybridization

In situ hybridization was performed as described (Humpel et al., 1993, Ullrich et al., 2011). We used the same well-established oligonucleotides as recently published for quantitative real time-PCR (Daschil et al., 2013, Schlick et al., 2010). Mice were decapitated, and the brains frozen under a CO_2_ stream, sectioned (14 μm) with a cryostat (Leica), and thawed onto slides (ProbeOn slides, Fisher Biotech, Austria). Oligonucleotides (5 pmol) were labeled at the 3′end with [α-^35^S]dATP using terminal deoxyribonucleotidyl transferase (Roche, Austria) and purified on Qiagen columns. The following oligonucleotides were used for in situ hybridization:

Ca_V_1.2 α_1_, forward 5′-cagcctgctctccacaga-3′; Ca_V_1.2 α_1_/1, reverse 5′- gggaatgtggtaggagaatgg-3′; Ca_V_1.2 α_1_/2, reverse 5′-caggtagcctttgagatcttcttc-3′; β_1_, reverse 5′-ctgcctccttccttaaggcttc-3′; β_2,_ reverse 5′-ctctcttgggtttcagagtcaaa-3′; β_3_, reverse 5′-acagtagctgacattggtcctcac-3′; β_4,_ reverse 5′-tgtctcattcgctgactctgtaat-3′; α_2_δ-1, reverse 5′-acagtccagtaaaccactgaatga-3′; α_2_δ-2, reverse 5′-cttcctgtccagcaggctct-3′; α_2_δ-3, reverse 5′-atttaatccctgggtactgtctga-3′; α_2_δ-4, reverse 5′-caaggaagtctctgcaaccag-3′; and PPT-A, reverse 5′-cattaatccaaagaactgctgaggcttggg-3′. Brain sections were hybridized overnight at 42 °C in a humidified chamber with 100 μL per section of the hybridization solution (50% formamide, 4× SSC, 0.02% polyvinylpyrrolidone, 0.02% Ficoll, 0.02% bovine serum albumin, 10% dextran sulfate, 0.5 mg/mL-sheared salmon sperm DNA, 1% sarcosyl (N-lauroyl sarcosine), 0.02 M NaPO_4_ (pH 7.0), and 50 mM dithiothreitol) containing 1 × 10^7^ CPM/mL probe. Sections were subsequently rinsed, washed 4 times (15 minutes each) in 1× saline sodium citrate at 54 °C, cooled to room temperature, dehydrated through 70%, 90%, and 99.9% ethanol, and subsequently air-dried. Sections were dipped in Kodak NTB photo emulsion, exposed for 5 weeks at −20 °C, developed, fixed, and counterstained with thioflavin S and DAPI (2 hours in the dark), and then mounted with Vectashield. Sections were stored in the dark at 4 °C until analysis. As a control, selected sections (incubated with radioactively labeled Ca_V_1.2 oligonucleotides) were incubated with 20× excess of unlabeled oligonucleotides.

### 2.3 Immunohistochemistry

Anesthetized mice were perfused transcardially with 20 mL of phosphate-buffered saline (PBS) and then with 50 mL of 4% paraformaldehyde (PAF) and 0.05% glutaraldehyde in 0.1 M phosphate buffer (PB, pH 7.4, without saline). Brains were dissected and washed in PB and subsequently stored in PB and 0.02% sodium azide at 4 °C until use. One half was used for electron microscopy (data not included and not shown) and the other half for confocal microscopy. One hemisphere was incubated overnight at 4 °C in PBS containing 20% sucrose, frozen in a CO_2_ stream, and stored at −80 °C until further processing. Brains were cut into coronal sections of 40 μm with a cryostat (Leica CM 1950). Immunohistochemistry was performed as described in detail (Daschil et al., 2013, Willis et al., 2010). Brain sections were washed with PBS and incubated in PBS and/or 0.1% Triton (T-PBS) for 30 minutes at 20 °C while shaking. After incubation, the sections were blocked in T-PBS, 20% horse serum (GIBCO Invitrogen), and/or 0.2% BSA (SERVA) for 30 minutes at 20 °C while shaking. Following blocking, brain sections were incubated with primary antibodies (GFAP, 1:2000, Millipore AB5541; Aβ, 1:250, Sigma A8978; collagen-IV, 1:500, Abcam, ab6586; alpha smooth muscle actin [αSMA], 1:1000; Novus Biologicals, NB300-978; Ca_V_1.2, 1:2000; Sigma C1603; substance P, 1:100, Bioss, bs-0065R) in T-PBS and/or 0.2% BSA over 2–3 days at 4 °C. The sections were then washed and incubated with fluorescent Alexa (-488, -546, or -647; Invitrogen-Life tech, Vienna, Austria) secondary antibodies (anti-rabbit for collagen-IV, substance P, Ca_V_1.2; anti-mouse for Aβ or anti-goat for αSMA) in T-PBS and/or 0.2% BSA for 1 hour at 20 °C while shaking. Finally, the sections were washed with PBS, some sections were counterstained with thioflavin S or thiazine red (both Sigma), and then mounted onto glass slides and cover-slipped with Mowiol 4-88 (Roth, Austria). Confocal microscopy was performed using an SP5 confocal microscope (Leica Microsystems, Wetzlar, Germany) with an HCX PL APO ×63 and/or 1.3 NA glycerol objective. Imaging was performed with an argon laser line for AlexaFluor 488, a DPSS561 nm laser for AlexaFluor 546 or thiazine red, and a HeNe 633 nm laser for AlexaFluor 647. Emission of each fluorophore was detected from 493 to 556 nm (AlexaFluor 488), 566 to 628 nm (AlexaFluor 546, thiazine red), and 638 to 750 nm (AlexaFluor 647). Images were acquired using the LAS AF acquisition software, version 2.1., and further processed with Huygens Deconvolution and Imaris V6.4 software.

### 2.4 Organotypic cortex brain slices

For cortex vibrosection cultures, 12-month-old adult APP_SweDI mice (developing extensive plaques) were used. All experiments conformed to Austrian guidelines on the ethical use of animals. Experimental steps were taken to reduce suffering and the number of animals used. Vibrosections were performed as described in detail (Ullrich et al., 2011). The animals were rapidly sacrificed, the brains dissected, and sagittally cut. The brains were glued onto the chuck of a water-cooled vibratome Leica VT1000A and triggered close to a commercial shave racer. Under aseptic conditions, 120-μm vibrosections were cut and collected in sterile medium. The organotypic vibrosections were carefully placed onto a sterile 0.4-μm pore membrane (Millipore HTTP02500), which was then placed into a 0.4-μm membrane insert (Millipore PICM03050) within a 6-well plate. Vibrosections were cultured in 6-well plates (Greiner) at 37 °C and 5% CO_2_ with 1.2 mL/well of the following culture medium: 50% MEM and/or HEPES (Gibco), 25% heat-inactivated horse serum (Gibco, Lifetechnologies, Austria), 25% Hanks' solution (Gibco), 2 mM NaHCO_3_ (Merck, Austria), 6.5 mg/mL glucose (Merck, Germany), 2 mM glutamine (Merck, Germany), and pH 7.2. Vibrosections were incubated for 4 weeks without or with 10 μM of the LTCC inhibitors nimodipine, nicardipine, nifedipine, and isradipine (all Sigma) or with 500 ng/mL substance P (SP acetate salt hydrate, Sigma S6883) or with both. Slices were then fixed for 3 hours with 4% PAF, then stained for collagen-IV (using Alexa-488), and the vascular density was counted in a 6 × 6 grid (Fig. 4) as described by us in detail (Moser et al., 2003).

### 2.5 Data analysis and statistics

**Fig. 1:**
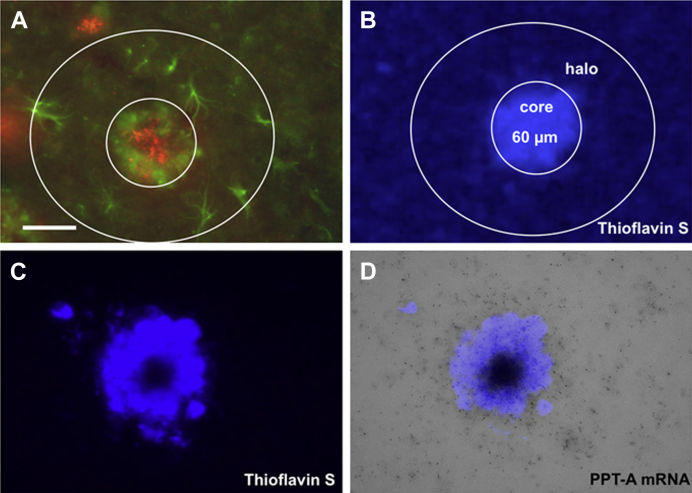
Immunohistochemical staining for reactive glial-fibrillary acidic protein (GFAP^+^) astrocytes (Alexa-488) around thioflavin S^+^ Aβ plaques (A) and definition of halo and core (B). A plaque was identified by thioflavin S staining under the fluorescence microscope and the plaque core (average diameter 60 μm) and the halo (3× the diameter of the core) are circled. In situ hybridization for PPT-A (C and D) mRNA in an 11-month-old APP_SL_ mouse. The plaques were identified by thioflavin S staining (C). Silver grains of PPT-A mRNA are centered directly above the thioflavin S-stained plaques (D). Scale bar in A = 40 μm. Abbreviations: Aβ, β-amyloid; mRNA, messenger RNA; PPT-A, preprotachykinin-A.

Plaques were identified by thioflavin S staining and photographed under a fluorescence microscope. Silver grains were visualized by means of a bright-field microscope. Using Openlab software (4.0.4) both pictures were overlaid to define the plaque core (in average 60 μm diameter) and the plaque halo (3× core, in average 180 μm diameter). The silver grains were counted by computer-assisted image analysis in the plaque core and in the halo (see Fig. 1). The total grain number in tg mice was determined by subtracting the corresponding cortical wt sections. Statistical analysis was performed by one way analysis of variance and subsequent Fisher least significant difference post hoc test. Statistical results were considered significant at *p* < 0.05.

## 3 Results

### 3.1 Detection of plaques in the Alzheimer mouse brain

In 11-month-old tg APP mice, a large number of Aβ plaques were detected. The number and localization were similar to those in previous studies (Daschil et al., 2013, Willis et al., 2010). No Aβ plaques were found in wt mice. Aβ plaques were identified by Aβ immunostaining (Fig. 1A), thioflavin S (Fig. 1B), thiazine red staining (Fig. 3), or Congo Red (not shown). The size of the plaques and the distance to the reactive astroglia allowed us to determine the core and halo diameter (Fig. 1). This distance is in full agreement with Serrano-Pozo et al. (2013), including reactive astroglia. Immunohistochemistry revealed that the plaques were surrounded by GFAP^+^ reactive astrocytes (Fig. 1A). Furthermore, we show that 62% ± 3% (n = 4) of all thiazine red^+^ plaques are associated with collagen-IV^+^ vessels (Fig. 4A). These vessels exhibited a thickness of 4.9 ± 0.5 μm.

### 3.2 Expression of PPT-A mRNA over plaques in the cortex

As a control, the neuropeptide PPT-A was tested for which we previously showed immunoreactivity in reactive astroglia in the plaque halo. Surprisingly, PPT-A mRNA was detected in the core and to a much lesser extent in the halo (Fig. 1C and D). This was also verified by semiquantitative analysis of silver grains in the core and halo (Table 1). To verify specificity of the staining, we point to a focused, strongly centered staining for PPT-A, whereas parts of the plaque core were negative (Fig. 1C and D).

**Table 1:** Semiquantitative analysis of silver grains for cortical mRNA expression of subunits of the L-type calcium channel and PPT-A

Probe	Wild type (ctx)	Sections analyzed	Transgenic animals (ctx)	
			Halo	Core
Sense (BG)	0.8 ± 0.12	12	1.0 ± 0.07	1.0 ± 0.08
PPT-A	0.9 ± 0.06	23	0.9 ± 0.08	3.1 ± 0.03^***^
Ca_V_1.2 α_1_	1.1 ± 0.1	43	1.0 ± 0.09	2.6 ± 0.1*
β_1_	0.8 ± 0.09	26	1.2 ± 0.08	1.9 ± 0.06
β_2_	0.9 ± 0.03	26	1.0 ± 0.09	5.8 ± 0.4^***^
β_3_	0.7 ± 0.1	14	1.1 ± 0.06	1.8 ± 0.1
β_4_	1.0 ± 0.1	24	1.4 ± 0.1	5.9 ± 0.3^***^
α_2_δ-1	0.8 ± 0.08	24	1.1 ± 0.2	5.4 ± 0.3^***^
α_2_δ-2	0.8 ± 0.01	29	1.1 ± 0.1	1.2 ± 0.1
α_2_δ-3	0.8 ± 0.01	13	0.8 ± 0.07	0.8 ± 0.05
α_2_δ-4	0.9 ± 0.04	15	0.6 ± 0.03	0.7 ± 0.05

The number of analyzed Alzheimer tg and wt brains was n = 4. The number of grains was counted by computer-assisted image analysis directly over the core (diameter 60 μm) or in the halo (180 μm diameter) in 11-month-old mice. Sense oligonucleotides defined the background (BG). Semiquantitative analysis showed that 31.1% ± 7.7% of all plaques were associated with vessels and expressed the calcium channel or PPT-A, the precursor for SP. Values are given as mean ± SEM grains/100 μm^2^. Statistical analysis was performed by one way ANOVA and subsequent Fisher least significant difference post hoc test (^*^p < 0.05; ^***^p < 0.001) by comparing against wild-type cortex (ctx) sections.

Significant values are indicated in bold.

Key: ANOVA, analysis of variance; PPT-A, preprotachykinin-A; SEM, standard error of the mean; SP, substance P.

**Table 2:** Angiogenetic effects of LTCC blockers and SP on plaque-associated vessels in cortical organotypic brain sections of adult Alzheimer mice

Treatment	Vessel density	*p* versus minus	*p*-values
Minus	76 ± 1.8 (16)	—	
SP	102 ± 5.8 (20)	***	
ISR	110 ± 1.6 (11)	***	
NIM	110 ± 2.2 (18)	***	
NIF	111 ± 2.2 (9)	***	
NIC	91 ± 2.4 (8)	**	
SP + ISR	113 ± 2.9 (11)	* versus SP	NS versus ISR
SP + NIM	123 ± 3.8 (12)	* versus SP	** versus NIM

Cortical vibrosections (120-μm thick) were cultured from 12-month-old APP_SweDI mice for 4 weeks without (minus), with 500 ng/mL SP, with 10 μM of the LTCC blockers isradipine (ISR), nimodipine (NIM), nifedipine (NIF), or nicardipine (NIC) or with both, SP and LTCC blockers for 4 weeks. Sections were then fixed and immunohistochemically stained for collagen-IV using Alexa-488. The vascular density was counted in a 6 × 6 grid at a ×10 magnification. Statistical analysis was performed by one way ANOVA and Fisher least significant difference post hoc test; *p < 0.05; **p < 0.01; ***p < 0.001.

Key: ANOVA, analysis of variance; LTCC, L-type calcium channel; SP, substance P.

### 3.3 Expression of LTCC subunits mRNA in hippocampus and cortex of wt controls

**Fig. 2:**
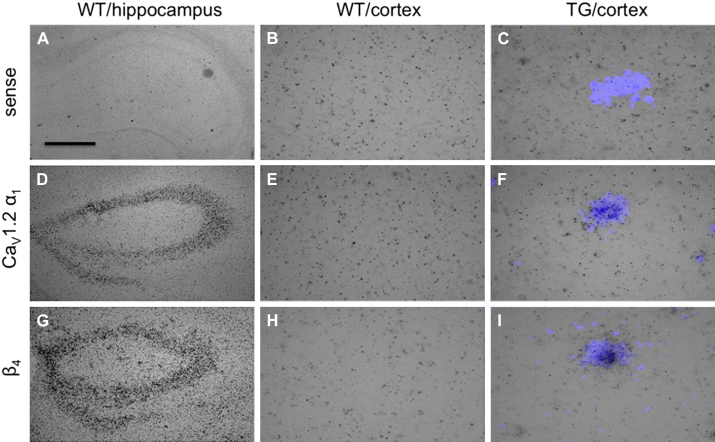
In situ hybridization for Ca_V_1.2 and auxiliary calcium-channel subunits in 11-month-old wt mice in the hippocampal formation (A, D, and G) and cortex (B, E, and H) and in tg mice in the cortex, where plaques were counterstained with thioflavin S (C, F, and I). The sense oligonucleotide revealed only background silver grains in the hippocampus (A) and cortex (B and C). The Ca_V_1.2 α_1_ (D–F) and β_4_ (G–I) antisense oligonucleotides-labeled neurons in the hippocampus (D and G), whereas only the background is seen in the wt cortex (E and H). Note, enhanced silver grain densities directly over the thioflavin S stained plaque core (F and I) for both probes. Scale bar in A = 210 μm (A, D, and G) and 70 μm (B, C, E, F, H, and I). Abbreviation: tg, transgenic; wt, wild type.

Several controls were performed to support the specificity of the hybridization signal. Because most of the LTCC subunits were strongly expressed in the hippocampal formation, it was especially evaluated in age-matched 11-month-old wt mice. Comparison of probes for different LTCC subunits, mRNA revealed a specific expression pattern with regard to distribution and expression levels in the hippocampal formation for Ca_V_1.2 α_1_ (Fig. 2D), β_4_ (Fig. 2G), and all other LTCC subunits (data not shown), except α_2_δ-4, which was not expressed. In addition, the α_2_δ-2 subunit mRNA was markedly expressed in the medial habenula (data not shown). Sense oligonucleotide probes revealed only background staining in the hippocampus (Fig. 2A). For Ca_V_1.2 α_1_, we used 2 different probes complementary for different regions within its gene, which revealed an identical expression pattern (data not shown). The expression of the LTCC subunit mRNAs in the parietal cortex of wt mice displayed a diffuse pattern (shown for α_1_ and β_4_ in Fig. 2E and H) and was slightly above the sense background (Fig. 2B; Table 1) and below the tg grain density (Fig. 2F and I; Table 1).

### 3.4 Expression of LTCC subunits mRNA in plaques in the cortex of tg mice

Background staining was especially evaluated in association with Aβ plaques and again sense probes displayed only background staining in tg cortex (Fig. 2C). In contrast to our hypothesis, Ca_V_1.2 α_1_ mRNA was not expressed in the plaque halo (Fig. 2F). Similarly, β_4_ mRNA was not expressed in the plaque halo (Fig. 2I), and none of the other β or α_2_δ subunits were found in the halo (data not shown). This was also verified by semiquantitative analysis of silver grains (Table 1). Surprisingly, the grain densities for Ca_V_1.2 α_1_ (Fig. 2F), β_2_, β_4_ (Fig. 2I), and α_2_δ-1 were significantly enhanced directly above the plaque core, whereas all other auxiliary subunits did not differ from wt mice (data not shown). Again, this was also verified by semiquantitative analysis of silver grains (Table 1). When a 20× excess of unlabeled oligonucleotides were added to Ca_V_1.2 α_1_ radioactive oligonucleotides, no grains were detected over plaques (data not shown). Semiquantitative analysis showed that 31.1% ± 7.7% of plaque-associated vessels express the calcium channel or substance P.

### 3.5 Confocal microscopy of vessels around Aβ plaques in the cortex

**Fig. 3:**
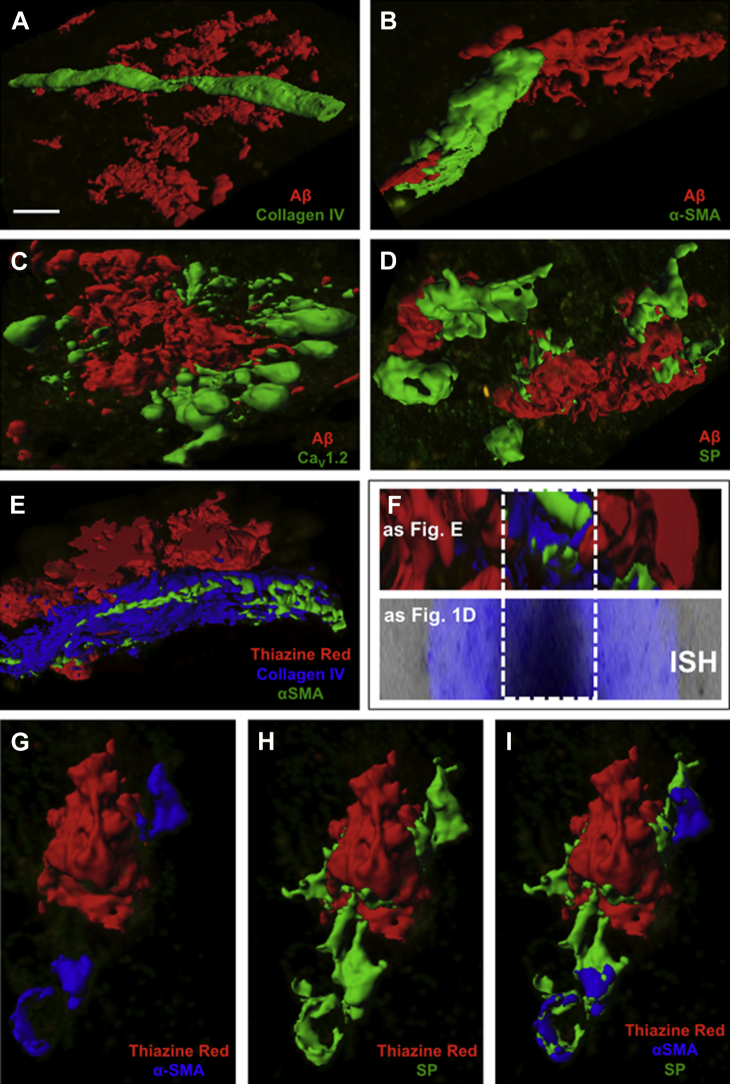
Confocal microscopy of vessels associated with Aβ plaques in the cortex of an AD mouse model. Plaques were stained either by Aβ immunohistochemistry (A–D) or with thiazine red (E–I). Brain vessels were identified with a collagen-IV staining (A and E) and pericytes using αSMA (B, E, F, G, and I). Both markers clearly labeled brain vessels associated with Aβ plaques (E). The α_1_ subunit of the Ca_V_1.2-LI was found mainly as a diffuse unclear pattern in an intermediate zone between the core and the halo (C), whereas SP was clearly found in cells close to the plaque (D). Comparison of sections stained with immunohistochemistry and sections labeled by in situ hybridization clearly shows that immunoreactive cells correspond to the silver grains directly in the plaque core (F). Triple staining illustrates that SP (H) co-stains with αSMA (G), demonstrating expression of SP in brain vessels associated with plaques (I). Scale bar in A = 10 μm (A–I). Abbreviations: αSMA, alpha smooth muscle actin; Aβ, β-amyloid; AD, Alzheimer's disease; SP, substance P.

Confocal microscopy confirms that collagen-IV^+^ brain vessels are associated with Aβ plaques in the AD mouse model (Fig. 3A). Furthermore, these vessels also contain pericytes expressing αSMA (Fig. 3B). Immunostaining for the α_1_ subunit of Ca_V_1.2 shows a diffuse unclear immunoreactivity, located mainly around the plaque in an intermediate zone between the core and the halo (Fig. 3C). Substance P-like immunoreactive cells are clearly associated with Aβ plaques (Fig. 3D). To perform triple staining with 2 antibodies, we stained plaques with thiazine red, which was as potent as the Aβ immunohistochemistry (Fig. 3E–I). Triple staining revealed that αSMA^+^ pericytes were indeed located in collagen-IV^+^ vessels associated with thiazine red plaques (Fig. 3E). Comparison of sections stained with immunohistochemistry and sections labeled by in situ hybridization clearly showed that immunoreactive cells correspond to the silver grains directly in the plaque core (Fig. 3F). Triple staining for pericytes, SP, and thiazine red plaques revealed that SP-immunostained cells were located together with pericytes in vessels associated with the plaque (Fig. 3G–I).

### 3.6 Angiogenesis in cortical organotypic brain slices

**Fig. 4:**
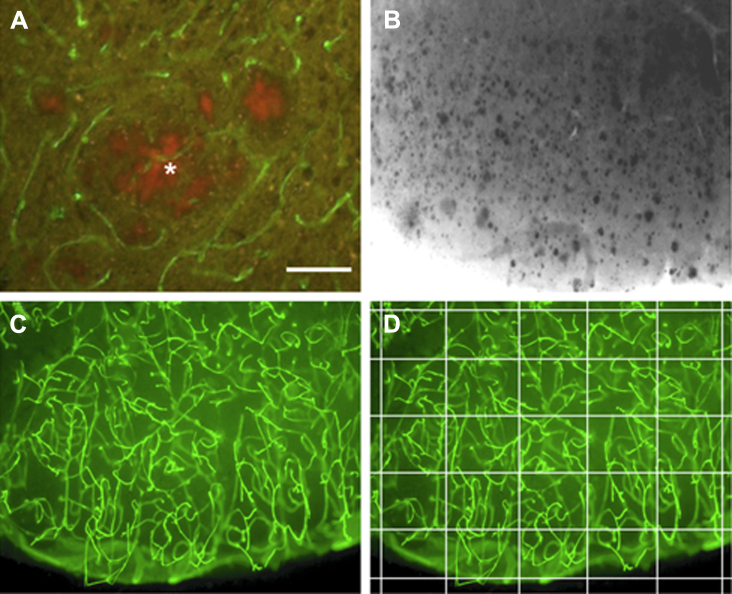
Collagen-IV^+^ brain vessels are directly associated with thiazine red^+^ plaques (A, *) of the APP_SweDI Alzheimer mouse model. In adult cortical organotypic brain vibrosections of an APP_SweDI Alzheimer mouse model, a high number of thiazine red plaques (shown in a black-white picture) is visible in the cortex after 4 weeks of incubation (B). These brain slices contain a dense network of collagen-IV^+^ (Alexa-488, green) brain vessels. Semiquantitative analysis of brain vessels was performed in a 6 × 6 grid by counting the crossings of the vessels (C, D). Scale bar in A = 25 μm, B–D = 85 μm.

Aβ plaques were largely associated with collagen-IV^+^ brain vessels (Fig. 4A). In adult slices of the APP_SweDI mice, Aβ plaques were visualized by thiazine red staining after culturing for 4 weeks (Fig. 4B). These slices exhibited a dense collagen-IV^+^ vascular network (Fig. 4C), which was quantified in a 6 × 6 grid (Fig. 4D) giving a network of 76 ± 1.8 (n = 16) crossings in control slices (Table 2). SP and all tested LTCC blockers induced angiogenesis in cortical slices (Table 2), which was slightly potentiated when both substances were applied (Table 2).

## 4 Discussion

In the present study, we show for the first time that mRNAs for an entire LTCC and PPT-A (the precursor for SP) were expressed above the Aβ plaque core in the cortex of Alzheimer mice. We further provide evidence that this calcium channel and SP are expressed on cortical brain vessels, and that both substances induce angiogenesis of plaque-associated vessels.

### 4.1 Aβ plaques in Alzheimer mice

The use of tg mice to study plaques is well-established, and we have extensively used these APP_SL_-overexpressing mice (Daschil et al., 2013, Willis et al., 2007, Willis et al., 2008, Willis et al., 2010). The plaques consist of a central core of highly aggregated Aβ peptides and a halo surrounding the plaques, with migrated reactive astrocytes and microglia. The most robust deficits occur in this halo around the plaques within a radius of approximately 20 μm, including local alterations in spine density, neuritic curvature, calcium dysregulation, and oxidative stress (Xie et al., 2013). We recently showed that these reactive astrocytes express the Ca_V_1.2 α_1_ subunit of the LTCC channel (Daschil et al., 2013). We also showed that these reactive astrocytes express different neuropeptides, such as SP (Willis et al., 2007). Thus, in the present study this neuropeptide should serve as a positive control.

### 4.2 Expression of PPT-A mRNA in Aβ plaques localized in the cortex

It was rather surprising to find PPT-A mRNA expression directly over the Aβ plaque core. PPT-A mRNA expression was localized in cells in the plaque core, possibly representing plaque associated vessels. No PPT-A mRNA expression was found in the halo of the plaque. Unfortunately, in situ hybridization does not allow co-localization studies with immunohistochemistry; however, our immunostainings provide evidence that SP is expressed on brain vessels associated with plaques. It is well-established that SP plays an important role in angiogenesis (Albrecht-Schgoer et al., 2012, Kohara et al., 2010, Pelletier et al., 2002, Seegers et al., 2003, Wiedermann et al., 1996), and, in fact, there is clear evidence that SP is expressed in endothelial cells (Milner et al., 2004, Stones et al., 1995), airway smooth muscle cells (Maghni et al., 2003), and pericytes (Maghni et al., 2003). Indeed, using organotypic brain cultures of adult AD mice, we show that SP exerts a strong and significant angiogenetic effect on brain vessels associated with plaques.

### 4.3 Plaques and vessels

There are clear indications that AD is linked to a vascular pathology (de la Torre, 2002, Humpel, 2011, Iadecola, 2004). Indeed, it has been reported that tg AD mice display structural changes in blood vessels and modified blood flow (Beckmann et al., 2003.). It is well known that 95% of dense plaques in Tg2576 and 85% in PSAPP mice are centered on vessel walls or in the immediate perivascular region (Kumar-Singh et al., 2005). An autopsy study of AD patients showed 60%–77% of Aβ plaques to be associated with blood vessels, however, 8%–13% of Aβ plaques are penetrated by vessels, and this vessel density is higher in the border zone of plaques (Kawai et al., 1990). In human sporadic AD patients, classic Aβ deposits cluster around large diameter blood vessels rather than capillaries (Armstrong, 2006). Impressive 3D electron microscopy clearly shows early Aβ deposits on blood vessels, including pompom-like and cube-shaped clumps clinging to brain microvessels in an AD mouse model (Meyer et al., 2008). Damage to the microvasculature begins before the onset of parenchymal Aβ plaques or vascular amyloid deposition (Meyer et al., 2008). Our present data are in line and show that approximately 60% of all Aβ plaques are associated with vessels.

### 4.4 Expression of LTCCs in the cortex

In our hands, in situ hybridization is a well-established and potent tool for visualizing mRNA expression at the cellular level (Humpel et al., 1993). Radioactive probes (^35^S) are more sensitive than nonradioactive (e.g., digoxigenin) probes. Although the use of riboprobes is highly specific and sensitive, we did not have access to such probes and therefore used commercial oligonucleotide probes. All these oligonucleotide probes have been well-characterized and applied in previous quantitative real time-PCR analyses (Daschil et al., 2013, Schlick et al., 2010). The present study detected high expression levels for most of the LTCC subunits, which is in line with a previous study (Schlick et al., 2010). Our data show that all probes were selective and specific for the LTCC subunits and displayed a well-known and established pattern for mRNA expression of Ca_V_1.2 α_1_ (Ludwig et al., 1997), the β_1–4_ auxiliary subunits (Ludwig et al., 1997), and the α_2_δ subunits (Cole et al., 2005) in the hippocampal formation.

### 4.5 Expression of LTCCs mRNA above the plaques in the cortex

We did not detect mRNA expression for Ca_V_1.2 α_1_ around the plaques in the halo, which is in contrast to our immunocytochemical study (Daschil et al., 2013, Willis et al., 2010). This mismatch between mRNA expression and protein has several potential explanations. The most likely reason is that the increased expression of the protein downregulates its own mRNA expression via negative feedback regulation (Groth et al., 2014). This is also in line with our recent findings, where we show (Daschil et al., 2014) that a chronic 3 week addition of Aβ_42_ to primary astrocytes down-regulates the mRNA expression of Ca_V_1.2 α_1_ subunits. In addition, it seems also possible that enhanced degradation and turnover may contribute to the down-regulation of the mRNA expression. Increased processing and alternative splicings including epigenetic mechanisms may also occur.

### 4.6 LTCC linked to the vascular system in Aβ plaques

It was rather unexpected to find silver grains for Ca_V_1.2 α_1_, and more pronounced for β_2_ and/or β_4_ and α_2_δ-1 subunits, located directly above the plaque core. As we rigorously controlled for specificity, our data indicate that cells located directly on the core of the plaques indeed express LTCC subunits. Thus, our data show that these cells located above the plaques express the Ca_V_1.2 α_1_/β_2_/β_4_/α_2_δ-1 profile. In fact, it is known that the Ca_V_1.2 channel with the α_1_/β_2_/β_4_/α_2_δ-1 profile is directly associated with the vascular system, for example, on pericytes. In the brain, the pericyte is an integral cellular component of the blood-brain barrier and together with endothelial cells, astrocytes, and neurons form the neurovascular unit. Pericytes have been proposed to play a role in functional activities of the blood-brain barrier, microcirculation, and macrophage activity, and it has been shown that calcium enters central nervous system pericytes via LTCCs (Kamouchi et al., 2004). The existence of LTCCs in smooth muscle cells of major cerebral arteries and arterioles is well-established (Abd El-Rahman et al., 2013, Alborch et al., 1995, Reimer et al., 2000). Furthermore, myocytes and pericytes are electrically coupled, transmitting calcium signals between arteriolar and venular networks dependent on gap junctions and calcium entry via LTCCs (Borysova et al., 2013). And, finally, also pericytes of the descending vasa recta express voltage-gated divalent currents that are carried by LTCCs (Zhang et al., 2010). It needs to be proven in further experiments that indeed pericytes express an LTCC and SP. Anyhow, our in vitro data clearly show that LTCCs directly induce angiogenesis of vessels associated with plaques in an AD mouse model and SP slightly potentiated this effect. The intracellular mechanisms are unknown; however, it has been shown that SP activated calcium-dependent nitric oxide in endothelial cells of the rabbit cornea (Ziche et al., 1994), which was necessary for neovascularization, a process, which could also occur in the brain.

### 4.7 Study limitations

The present study has some methodological limitations. (1) Combination of the radioactive in situ hybridization method and immunocytochemistry on the same section did not yield reliable results. Therefore, we cannot provide direct evidence for localization of LTCC and SP mRNA and/or protein on the same section. (2) Specific antibodies for various LTCC subunits are not available, which is a major drawback for the cellular and subcellular localization of these proteins. A detailed subunit composition of LTCC subunits could not be performed because of the lack of specific antibodies except Ca_V_1.2 and β_4_. (3) The cross-sectional design of the study prevented us from clarifying whether the expression pattern is time dependent and whether it is related to the Aβ load. However, the present study is in line with a previous study using quantitative real time PCR. It also did not reveal longitudinal changes in the expression pattern of LTCC subunits. (4) We cannot provide any direct evidence that LTCCs are directly expressed on pericytes. (5) As we show angiogenesis of collagen-IV^+^ vessels, we cannot exclude, although very unlikely, that LTCC blockers or SP just upregulated collagen-IV expression in the brain vessels, rather than inducing formation of new tubes.

In conclusion, our data show that brain vessels are closely associated with Aβ plaques in the cortex of an AD mouse model. These cells express an LTCC and SP, suggesting a potent role in angiogenesis and vessel plasticity around Aβ plaques in AD.

## Disclosure statement

None of the authors have actual or potential conflicts of interests.
